# Heterogeneous porous biochar-supported nano NiFe_2_O_4_ for efficient removal of hazardous antibiotic from pharmaceutical wastewater

**DOI:** 10.1007/s11356-023-30587-5

**Published:** 2023-11-06

**Authors:** Ahmed B. Azzam, Yousif A. Tokhy, Farida M. El Dars, Ahmed A. Younes

**Affiliations:** Chemistry Department, Faculty of Science, Helwan University, Ain Helwan, Cairo, 11795 Egypt

**Keywords:** Ciprofloxacin (CIP), Nickel ferrite (NiFe_2_O_4_), Biochar (BC), Removal

## Abstract

Due to the dual issues of antibiotic resistance and bioaccumulation toxicity, antibiotics are ubiquitously present in aquatic environments, and this is causing serious concern. Herein, novel nickel ferrite (NiFe_2_O_4_) nanoparticles were successfully loaded onto activated biochar (BC) derived from banana peel (BP) to obtain magnetic nanocomposite (BC-NiFe_2_O_4_) as an effective biosorbent for the ciprofloxacin antibiotic (CIP) elimination from pharmaceutical effluent. A facile co-precipitation approach was utilized to construct the heterogeneous BC-NiFe_2_O_4_. The synthesized materials were systematically characterized using techniques such as XRD, FE-SEM, EDX, HR-TEM, BET, FTIR, and XPS. In addition, the magnetic measurements indicated the ferromagnetic behavior of the BC-NiFe_2_O_4_ sample. The influencing factors (i.e., pH, contact time, initial concentration, dose of adsorbent, ions interference, and solution temperature) of the adsorption process were also well studied. The adsorption capacity of the BC-NiFe_2_O_4_ heterostructure was 68.79 mg g^−1^ compared to the BC sample (35.71 mg g^−1^), confirming that the loading of magnetically NiFe_2_O_4_ nanoparticles onto the surface of porous biochar enhanced its stability and adsorption performance for CIP removal, wherein the metal-antibiotic complex has a significant effect for the removal of CIP. Moreover, the Langmuir adsorption isotherm and the pseudo-second-order model displayed a good fit for the experimental data. The values of △*H*° and △*G*° revealed that the adsorption process was endothermic and spontaneous. The coordination affinities, π-π stacking, and H-bonding interactions play a more critical role in the adsorption mechanism that confirmed by FTIR and XPS analysis. To study the stability of BC-NiFe_2_O_4_ nanocomposites, desorption and recycling studies were investigated. The results revealed that after three cycles, no significant loss in removal efficiency was detected, reflecting the stability and reusability of the prepared BC-NiFe_2_O_4_ nanocomposite.

**Supplementary Information:**

The online version contains supplementary material available at 10.1007/s11356-023-30587-5.

## Introduction

The emerging contaminants from pharmaceuticals are spread throughout all environmental matrices and therefore must be remediated before their discharge into chemical treatment plants (Ashiq et al. 2019; Aydin et al. 2019; Krasucka et al. 2021; Yao et al. 2021; Apreja et al. 2022; Okeke et al. 2022; Mittal et al. 2023). One of the medicinal products is the antibiotics family which are essential in the treatment of human and animal contagious diseases. Nonetheless, even at trace levels, there is widespread worry about their toxicity for non-target organisms (Li et al. 2019). One of the most widely used antibiotics for treating bacterial infections and stimulating animal growth is ciprofloxacin (CIP), a broad-spectrum antibiotic from the fluoroquinolone family that was classified by the World Health Organization (WHO) as a high-priority emerging organic pollutant (Egbedina et al. 2021). As a consequence of its widespread use, inadequate wastewater treatment and environmental discharge, CIP has been detected in a wide range of systems, including soil, sediment, drinking water, living tissues, and ground waters. This has sparked worries about their toxicity (Li et al. 2019; Dutta and Mala 2020). The existence of CIP in water ecosystems is particularly harmful to aquatic organisms (Li et al. 2019; Egbedina et al. 2021). The amount of ciprofloxacin measured in surface and groundwater is less than 1 g L^−1^ (Lins et al. 2020; Chaves et al. 2022). CIP concentrations in wastewater from healthcare and medication facilities may reach 150 g L^−1^ and 50 g L^−1^, respectively (Lins et al. 2020). Because of their high stability and wide dispersion, these antibiotics are non-dissolving long-range pollutants that must be removed before disposal. Fig. S1 depicts the life cycle of pollutants that emerge from healthcare facilities and pharmaceutical industries and diffuse to receptors. These antibiotics are highly detectable in water during their diffusion (Bhagat et al. 2020). To rapidly remove antibiotics from wastewater, efficient and commercially viable approaches should be developed. Reverse osmosis (Alonso et al. 2018), ion exchange (Wang et al. 2010, 2016; Bajpai and Bhowmik 2011), ultrafiltration (Palacio et al. 2018; Banerjee et al. 2019; Bhattacharya et al. 2019), ozonation (Gomes et al. 2017), advanced oxidation (Mondal et al. 2018), sedimentation, catalytic degradation (Gholami et al. 2020), and solvent extraction (Alaa El-Din et al. 2018; Akpomie and Conradie 2020) are some of the methods utilized to remove CIP from wastewater. The major disadvantages of these techniques include their high cost, hazardous by-product production, and difficult operability (Chen et al. 2021). Adsorption is a promising technique when compared to the prementioned methods because of its easy modification, reduced consumption, and low cost (Sun et al. 2019; Dai et al. 2020). Banana peel is a low-cost biosorbent with high adsorption behavior due to the number of adsorption active sites and natural components such as lignin and cellulose that are efficient in eliminating hazardous substances (Hashem et al. 2020). Biomass undergoes a variety of thermochemical processes, such as hydrothermal carbonization, and pyrolysis, to increase its adsorption capacity and kinetics (Wang et al. 2020). Biochar is produced from natural biomaterials by pyrolysis technique and has numerous applications in catalysis and water remediation due to its pore size and suitable physical and chemical properties (Lee et al. 2017). Biochar has received a lot of attention lately as a cheap and highly effective adsorbent due to its large pore volume, suitable physicochemical features, and ease of modification (Ouyang et al. 2020; Masrura et al. 2021). However, the low density of banana peel biochar implies insufficient dispersion in water. Consequently, the interaction ability of the soluble pollutants decreases, leading to a limited adsorption power. To overcome the separation difficulty, banana peel biochar is utilized as an accommodator for several magnetic nanoparticles. Due to their high adsorption effectiveness and ease of magnetic separation, magnetic nickel ferrite/biochar composites are a type of promising adsorbents; however, their synthesis involves with high costs and secondary environmental effects.

In this study, the highly magnetic NiFe_2_O_4_ nanoparticles were successfully imbedded on the surface of the biochar made from the banana peel biomass through a facile, rapid, and low cost co-precipitation method. The magnetic nanocomposite (BC-NiFe_2_O_4_) was used for the removal of ciprofloxacin antibiotic (CIP) from pharmaceutical wastewater. The metal antibiotics complex plays an important role in CIP removal. In addition, none of the available studies have utilized BC-NiFe_2_O_4_ heterogeneous structure for ciprofloxacin (CIP) removal and the sorption mechanisms of CIP were also not properly evaluated. Therefore, the interactions between bio-modified inorganic–organic waste and pharmaceutical sorption were investigated. Adsorption batch methodology was used to evaluate the influence of operational parameters on CIP removal, including solution pH, adsorbent dose, initial concentration of CIP, solution temperature, and interfering ions. The adsorption performance of porous biochar (BC) and BC-NiFe_2_O_4_ was examined using kinetic parameters, rate-controlling mechanisms, isotherms, and thermodynamics studies. Moreover, industrial pharmaceutical wastewater sample was studied. Potential adsorption mechanisms were proposed and verified using FTIR and XPS analyses.

## Experimental

All materials and the characterization methodologies were listed in the electronic supplementary information (ESI).

### Porous biochar preparation and its surface modification

Banana peel waste (BP) was used to produce activated biochar (BC) as previously indicated (Fig S2) (Azzam et al. 2022). In detail, banana peels (BPs) were washed with distilled water several times to remove any impurities and adhere contaminants. The cleaned banana peels were then chopped into 2*2 cm pieces and dried at 105 °C for 4.0 h until a constant weight was achieved (Munagapati et al. 2018; Chakhtouna et al. 2021). Using a laboratory sieve, the dried BPs were crushed to the proper size of 150 µm. To construct porous biochar (BC), banana peel powder was activated using H_3_PO_4_ by uniformly mixing 5 g of powdered BP with 10 mL of 30% H_3_PO_4_ for 30 min. Then, the mixture was calcined in air for two hours at 300 °C to carbonize the components of the mixture. The biochar was washed several times with distilled water to neutralize the solution's pH and eliminate any impurities. The produced biochar was then dried in an oven for 6 h at 80 °C.

### *Synthesis of biochar loaded NiFe*_*2*_*O*_*4*_* (BC-NiFe*_*2*_*O*_*4*_*)*

Through a facile co-precipitation method, the highly magnetic NiFe_2_O_4_ nanoparticles were successfully imbedded on the surface of the biochar made from the banana peel biomass. Firstly, NiFe_2_O_4_ nanoparticles were prepared as follows, 0.1 g of nickel chloride and 0.57 g of ferric chloride were dissolved in 50 mL of distilled water for 30 min with continuous stirring at 50 °C. Sodium hydroxide (1.00 M) was added to the mixture to increase the pH above 12. Oleic acid was added to the mixture (1–2 drops) as a surfactant. After that, the mixture was stirred well for 30 min at 80 °C. The produced sample was washed with distilled water and ethanol to remove undesirable impurities and residual surfactant. The product was then centrifuged and dried overnight at 80 °C.

To construct BC-NiFe_2_O_4_ nanocomposites, 1.00 g of the obtained biochar was dispersed in 40 mL of deionized water for 10 min (solution A). The nickel and iron precursors were individually blended in 10 mL of deionized water, and the mixture was magnetically stirred well for 30 min at 50 °C (solution B). After carefully adding solution B to solution A, a drop of 1.00 M aqueous NaOH solution was added. The mixture was then heated for 45 min at 70 °C under magnetically stirring. After the reaction was completed, the mixture was centrifuged, washed with distilled water to neutralize the pH, and dried at 80 °C (Sagadevan et al. 2018; Chakhtouna et al. 2021).

### Batch adsorption experiments

To determine the factors affecting the ability of the adsorbents to remove CIP, batch adsorption studies were conducted in 50-mL volumetric bottles at 25 °C. The following variables; pH (2.0–10.0), contact times (0–120 min), CIP concentrations (20–150 mg L^−1^), adsorbent dosage (5.0–100.0 mg), and the temperature (298–328 K) were investigated. The CIP concentration was directly measured at λ_max_ 275 using a UV/Vis spectrophotometer (Mahmoud et al. 2021). The following equations were used to determine the adsorption performance *q*_e_ (mg g^−1^) and CIP removal efficiency (%):

1 $$R\mathrm{emoval effeciency \%}=\frac{{C}_{0}-{C}_{e}}{{C}_{0}}\times 100$$

2 $${q}_{e}(\mathrm{mg}/\mathrm{g})=\frac{{(C}_{0}-{C}_{\mathrm{e}})V}{W}$$

*C*_0_ represents the initial CIP concentration, *C*_*e*_ represents the CIP concentration at equilibrium (mg L^−1^), *V* represents the volume of the solution in liters, and *W* represents the weight of the adsorbate in grams (Avcı et al. 2019; Peñafiel et al. 2021).

## Results and discussions

### XRD analysis

**Fig. 1:**
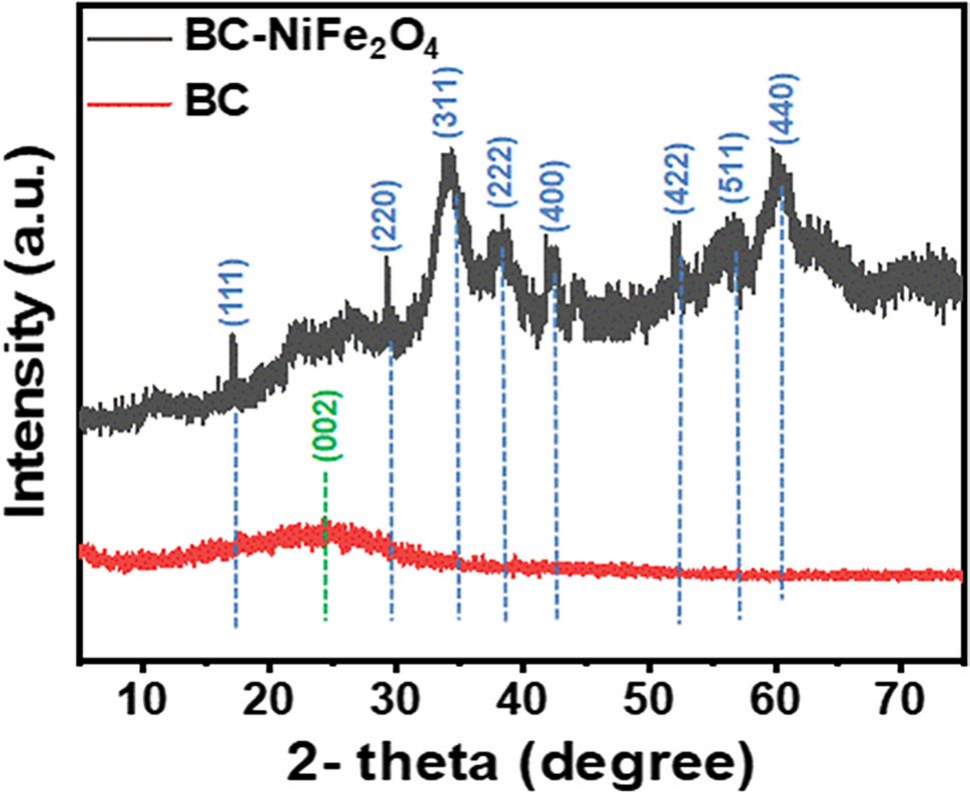
X-ray diffraction patterns of BC and BC-NiFe_2_O_4_ samples

The X-ray diffraction (XRD) patterns of BC and BC-NiFe_2_O_4_ nanocomposite were investigated as shown in Fig. 1. The BC diffraction pattern shows a characteristic a convex peak at 2θ ≈ 23° with a low dispersal angle, corresponding to graphitic-like microcrystals in biochar as identified by the JCPDS Card No. 46–1045 (Ding et al. 2014; Gupta and Gupta 2016; Tong et al. 2018; Patel et al. 2021a). This broad pattern suggests the amorphous properties of high porosity BC. In contrast, loading NiFe_2_O_4_ nanoparticles causes variations in biochar properties. The XRD pattern of the BC-NiFe_2_O_4_ sample exhibits distinct diffraction peaks at specific angles. These peaks are observed at 18.32° (111), 30.18° (220), 35.10° (311), 37.33° (222), 45.08° (400), 53.73° (422), 57.49° (511), and 63.13° (440), which are attributed to the presence of NiFe_2_O_4_ nanoparticles, as indicated by the JCPDS card No. 75–0035 (Hong et al. 2012; Livani et al. 2018; Sabaa et al. 2022). The crystallinity patterns of BC became more sharper after loading with NiFe_2_O_4_ nanoparticles. This findings implied that nickel ferrite (NiFe_2_O_4_) nanoparticles were successfully loaded onto porous biochar (BC).

### Surface morphology

**Fig. 2:**
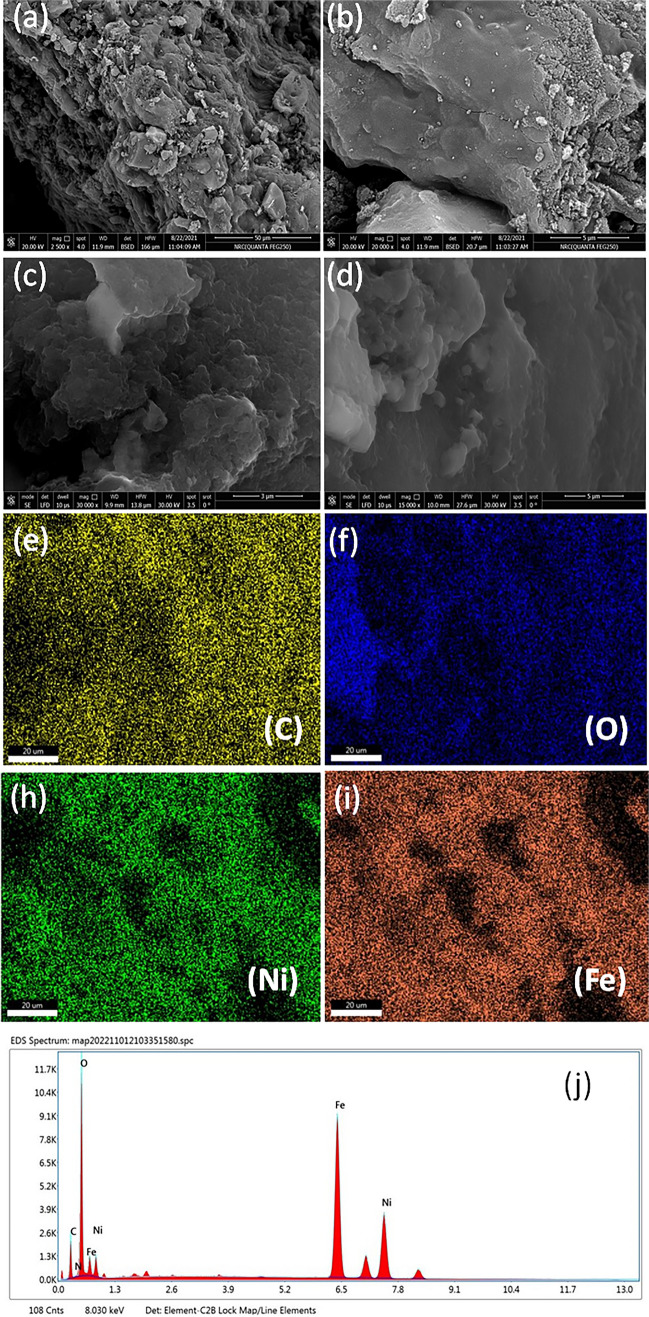
FE-SEM images BC (**a**, **b**), BC-NiFe_2_O_4_ (**c**, **d**); elemental mapping of BC-NiFe_2_O_4_ showing the uniform distribution of C, O, Ni, and Fe (**e**–**i**); EDX of BC-NiFe_2_O_4_ (**j**)

FE-SEM showed the structural morphology of the BC and BC-NiFe_2_O_4_ adsorbents. The biochar (BC) surface displayed multilayer porosity and dispersed particles with various shapes, as shown in (Fig. 2a, b). After NiFe_2_O_4_ loading, there are nano spherical particles deposited on the surface of BC sheets (Fig. 2c, d). According to the elemental mapping images of (Fig. 2e–i), the C, O, Ni, and Fe elements were distributed uniformly throughout the BC-NiFe_2_O_4_ sample. The BC-NiFe_2_O_4_ EDX spectrum, shown in Fig. 2j, demonstrates that spherical NiFe_2_O_4_ was successfully loaded onto the surface of BC.

**Fig. 3:**
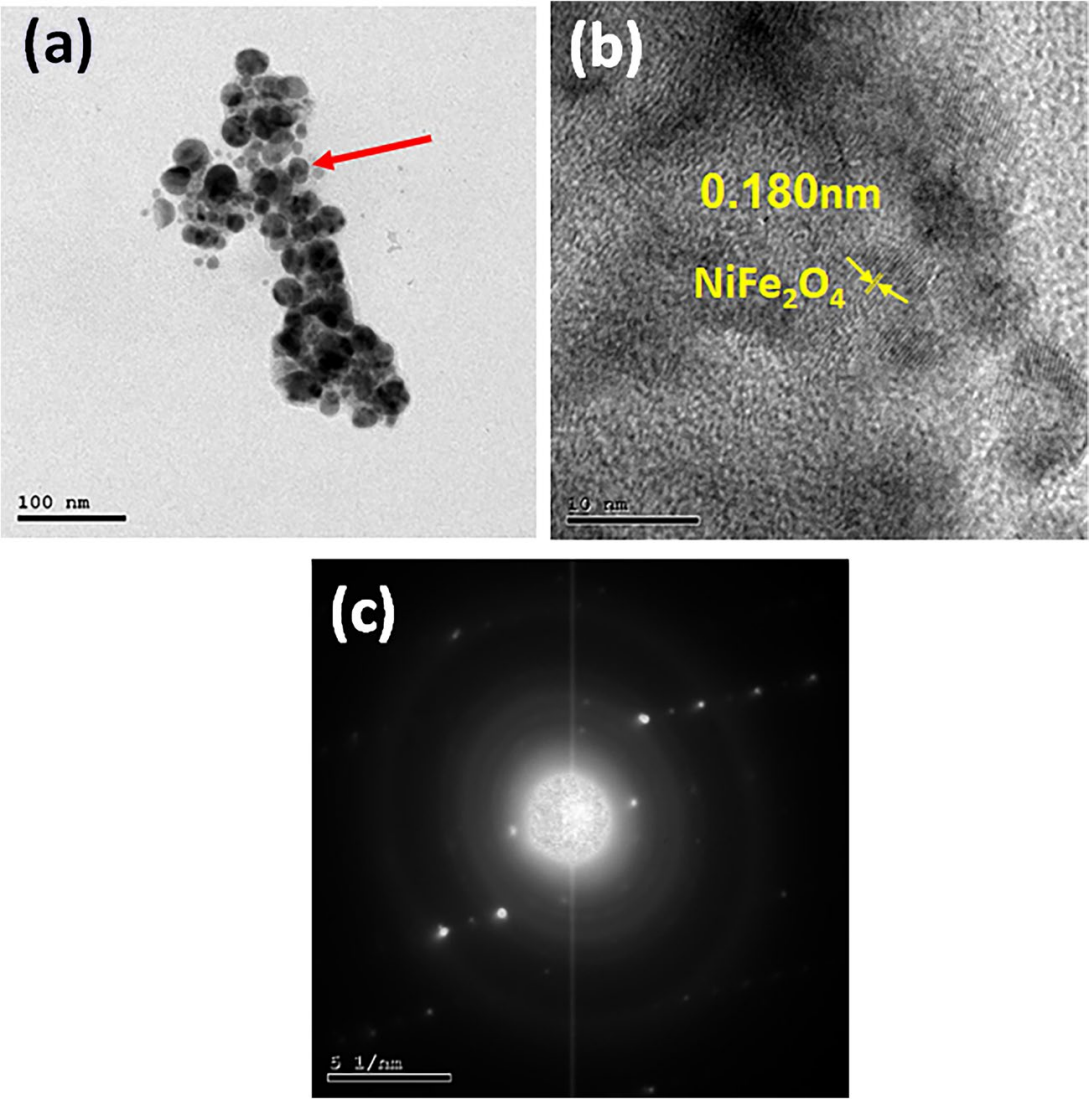
HR-TEM images of BC-NiFe_2_O_4_ nanocomposite

HR-TEM was further conducted on the structure of the BC-NiFe_2_O_4_ nanocomposite. As shown in Fig. 3a, b, the BC-NiFe_2_O_4_ heterostructure has a clear irregular spherical structure with a diameter of 5–30 nm. The existence of lattice patterns (*d*-space = 0.180 nm) with lattice plane (220), related to NiFe_2_O_4_ nanoparticles (Fig. 3b), proves that the combination of BC and NiFe_2_O_4_ was successful. The appearance of bright points stacked on a ring pattern revealed by SAED confirms the polycrystallinity of BC-NiFe_2_O_4_ nanocomposites (Fig. 3c).

### BET surface area and adsorption–desorption isotherm

The porosity characteristics of BC and BC-NiFe_2_O_4_ were investigated by studying the N_2_ adsorption–desorption isotherms and BET surface. The N_2_ isotherms of BC and BC-NiFe_2_O_4_ was illustrated in Fig. S4. According to IUPAC, type IV isotherms in the range of 0.5–1.0 P/P_0_ with obvious H_3_ hysteresis loops can be observed, indicating the formation of plentiful mesoporous structure (Sun et al. 2023).

As shown in Table 1, the specific surface area of BC (732 m^2^ g^−1^) was dramatically decreased after the loading of NiFe_2_O_4_ nanoparticles (252 m^2^ g^−1^) because of the blockage of BC pores with NiFe_2_O_4_ nanoparticles, confirming the successful formation of BC-NiFe_2_O_4_ heterostructures. Also, the total pore volume (*V*_*T*_*)* reduced from 0.553 to 0.201 cm^3^ g^−1^. Therefore, the external surface of BC became rougher and more heterogeneous after NiFe_2_O_4_ nanoparticles were loaded. In addition, the average pore size (*D*_*A*_*)* of BC and BC-NiFe_2_O_4_ samples was at 1.51 and 1.59 nm, respectively, showing that the two adsorbents are porous materials.

**Table 1:** Morphological properties of BC and BC-NiFe_2_O_4_

Material	V_*T*_ (cm^3^ g^−1^)	*D*_*A*_ (nm)	S_*BET*_ (m^2^ g^−1^)
BC	0.553	1.510	732.750
BC-NiFe_2_O_4_	0.201	1.598	252.255

### Magnetic properties

The magnetic properties of NiFe_2_O_4_ and BC-NiFe_2_O_4_ nanocomposite were investigated, and the results are presented in Table S1. The magnetic hysteresis curve (M-H) of the BC-NiFe_2_O_4_ nanocomposite was recorded at a temperature of zero Kelvin (0 K), as depicted in Fig. S5. The M-H plot displayed a characteristic hysteresis loop, indicating the ferromagnetic behavior of the BC-NiFe_2_O_4_ sample. This facilitates their recovery by using an external magnetic field. On the magnetic hysteresis curve, a saturation magnetization value of 10.02 emu g^−1^ was observed for the BC-NiFe_2_O_4_ nanocomposite which lower than pure NiFe_2_O_4_ nanoparticles (32.56 emu g^−1^). This decrease can be attributed to the presence of the banana peel biochar. It is worth noting that the majority of the composite material consists of banana peel biochar (BC). Consequently, the total magnetization may have been affected by the biochar’s nanomagnetic characteristics, which would indicate a lower saturation magnetization value in the BC-NiFe_2_O_4_ nanocomposite.

### Adsorption performance evaluation

#### Influence of solution pH

Ciprofloxacin adsorption is dependent on pH-based speciation. The results showed the superiority of the CIP adsorption onto BC and BC-NiFe_2_O_4_ at pH 6.0. This finding could be explained by the CIP (pKa_1_ = 6.09, pKa_2_ = 8.64), meaning that it exists as protonated (pH < 6.09), deprotonated (pH > 8.64), neutral and zwitterionic forms (6.09 > pH < 8.64) (Patel et al. 2021b). Both zwitterionic and neutral ciprofloxacin occur between 6.09 and 8.64, with the zwitterion dominant. Its piperazine secondary aliphatic amine form is protonated at pH 6.09. The secondary amine of the zwitterion’s piperazine group or the carboxyl group of the neutral form deprotonate at a solution pH > 8.64 to produce anionic ciprofloxacin. The maximum removal ratio was at pH 6.0, where the CIP is predominantly as neutral and zwitterionic ciprofloxacin forms. All of these forms have the ability for electron donor–acceptor interaction, π-π stacking, and H-bonding. Further, BC and BC-NiFe_2_O_4_ have a point of zero charge (pH_pzc_) of 6.12 and 7.23, respectively. At pH > pH_pzc_, the removal % declined slightly due to charge-charge repulsions with negatively charged of adsorbents. When the pH < pH_pzc_, the CIP removal reduced due to a repulsive interaction with positively charged of adsorbents.

#### Influence of contact time and initial concentration

**Fig. 4:**
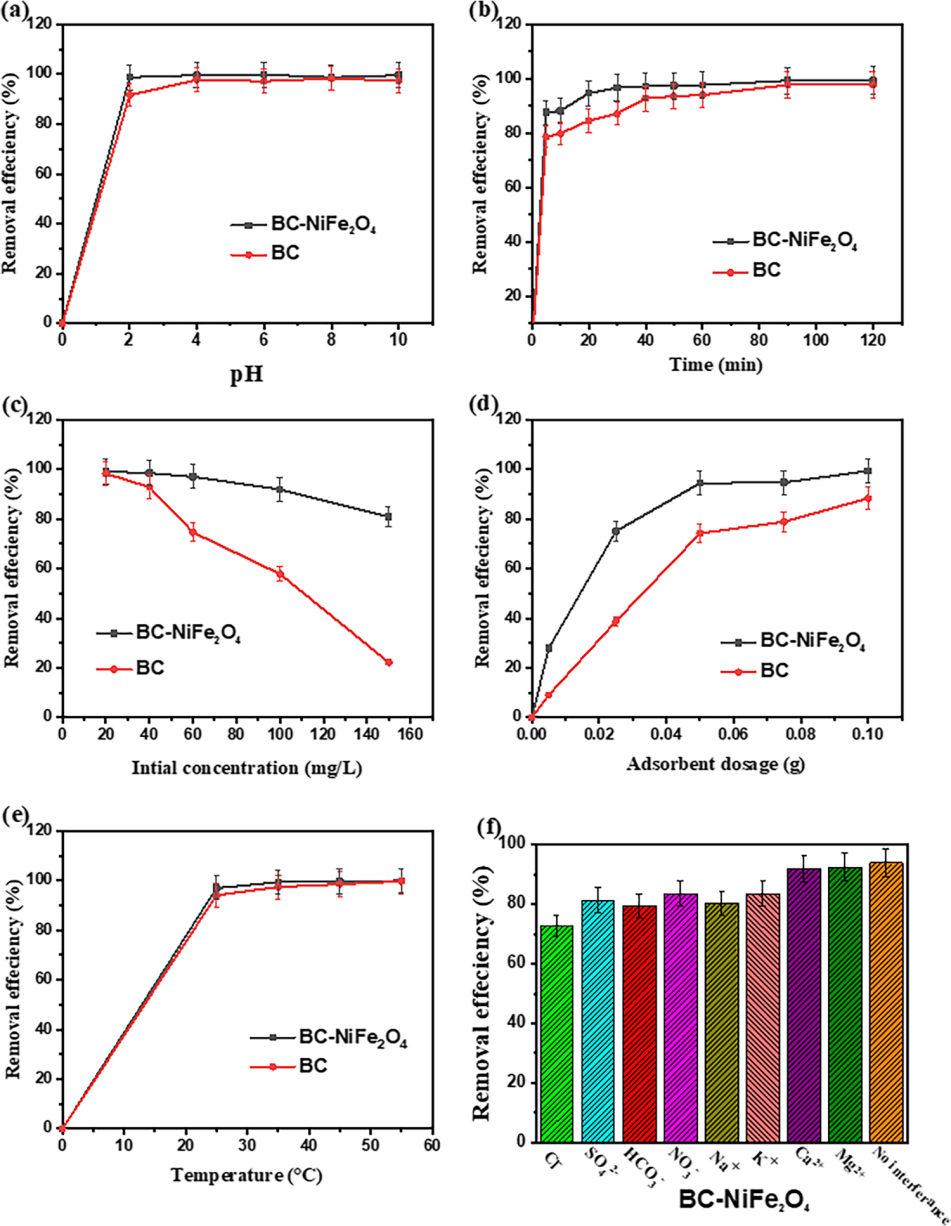
Effect of **a** initial pH, **b** contact time, **c** initial CIP concentration, **d** adsorbent dosage, **e** solution temperature, and **f** interfering ions of CIP adsorption onto BC and BC-NiFe_2_O_4_ nanocomposite. ([CIP] = 40 mg L^−1^ (except **a**, **c**) (**a**); [CIP] = 20–150 mg L^−1^ (**c**); [adsorbent dosage] = 2 g L^−1^ (except **d**); *T* = 298 K (except **e**); without pH adjustment (except **a**))

Figure 4b depicts the quantity of CIP adsorbed as a function of time. The removal efficiency of CIP onto BC and BC-NiFe_2_O_4_ samples was initially faster due to the presences of more available active sites on the surfaces of adsorbents. Almost 79.9% and 88.24% of the CIP were removed using BC and BC-NiFe_2_O_4_ within the first 10 min, respectively. Moreover, the adsorption percentages increased gradually until they reached equilibrium on BC and BC-NiFe_2_O_4_ after 90 min. Accelerated kinetics in the first 10 min are considered to be responsible for the increase of available active sites on the surface BC and BC-NiFe_2_O_4_ which gradually decreased over time due to the accumulation of CIP molecules on their surfaces reducing the uptake percentage (Tran et al. 2019). This phenomenon was caused by the general ionic migration of CIP into active pores and binding sites, which continued till all sites were filled (Yan et al. 2021).

On the other side, the initial CIP concentration has a significant influence on the adsorption performance of BC and BC-NiFe_2_O_4_ adsorbents. The impact of the initial CIP concentration was investigated between 20 and 150 mg L^−1^ at conditions (100 mg adsorbent; pH is 6; temperature 25 °C), as shown in Fig. 4c). The adsorption percentage of BC and BC-NiFe_2_O_4_ samples reduced from 98.2 to 22.2% and from 99.1 to 80.8%, respectively, when the initial concentration of CIP was raised from 20 to 150 mg L^−1^. The active sites on the surface adsorbents became unavailable as CIP concentrations increased. Comparing these results to our previous study on BC-NiS, they exhibited greater removal percentages (Azzam et al. 2022).

#### Influence of the adsorbent dose

Figure 4d illustrates the effect adsorbent dosage of BC and BC-NiFe_2_O_4_ onto the removal efficiency %. The increment in the dose of adsorbent from 5 to 100 mg enhanced the removal efficiency from 27.92 to 99.31% for BC-NiFe_2_O_4_. This increments have explained by providing more adsorption sites onto the surface of adsorbent (Peng et al. 2015; Abd El-Monaem et al. 2023; Omer et al. 2023).In addition, loading of NiFe_2_O_4_ into BC increased removal % to 99.31% compared to BC (95.82%).

#### Influence of solution temperature

The impact of temperature on the adsorption behavior of CIP onto BC and BC-NiFe_2_O_4_ was investigated from 298 to 328 K. As shown in Fig. 4e, raising the temperature improved removal efficiency % of CIP. The availability of more adsorption sites and increasing in CIP ions motion toward adsorbents accounts for the observed rise in removal percentages upon increasing the temperature. Therefore, the penetration of CIP molecules into the adsorbent pores is improved by raising the temperature (Kumari et al. 2020; Eltaweil et al. 2023).

#### Effect of ions interference

The effect of interfering ions on the CIP adsorption onto BC-NiFe_2_O_4_ heterostructure was investigated in the presence of several cations and anions. The presence of cations, e.g., Na^+^, K^+^, Mg^2+^, and Ca^2+^, slightly effect on CIP adsorption onto BC-NiFe_2_O_4_ but anion species such as Cl^−^, HCO_3_^−^, NO_3_^−^, and SO_4_^2−^ declined the removal efficacy (%) of CIP from 98.4 to 75.2, 79.5, 83.5, and 81.3%, respectively. This inhibitory effect was ascribed to competition for available sites on the BC-NiFe_2_O_4_ surface.

### Adsorption kinetics

**Fig. 5:**
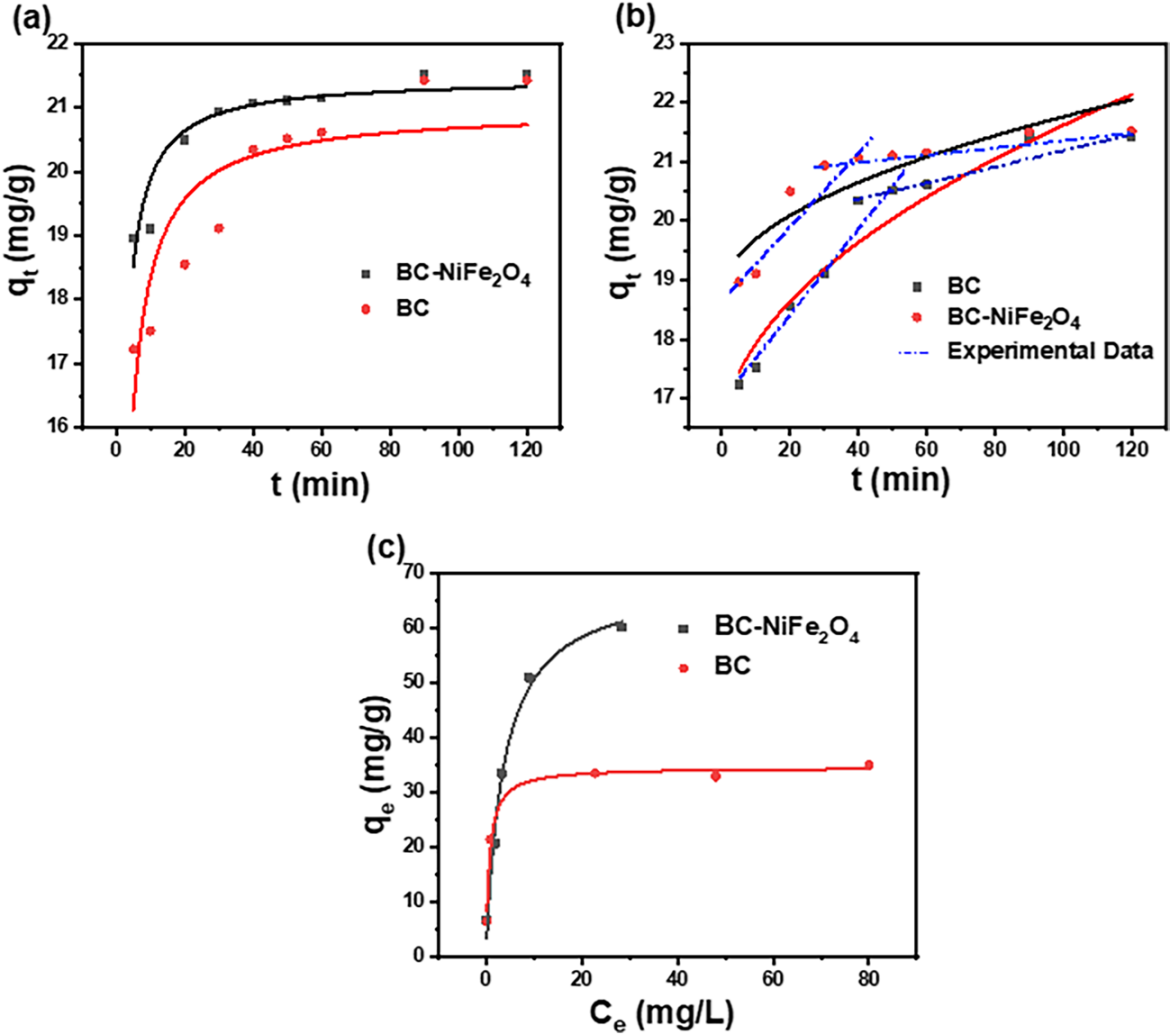
**a** Pseudo-second-order plot; **b** intra-particle diffusion model; **c** Langmuir adsorption isotherms plot onto BC and BC-NiFe_2_O_4_ adsorbents. ([CIP] = 40 mg L^−1^ (**a**, **b**); [CIP] = 20–150 mg L^−1^ (**c**); [adsorbent dosage] = 2 g L^−1^, *T* = 298 K; without pH adjustment)

**Fig. 6:**
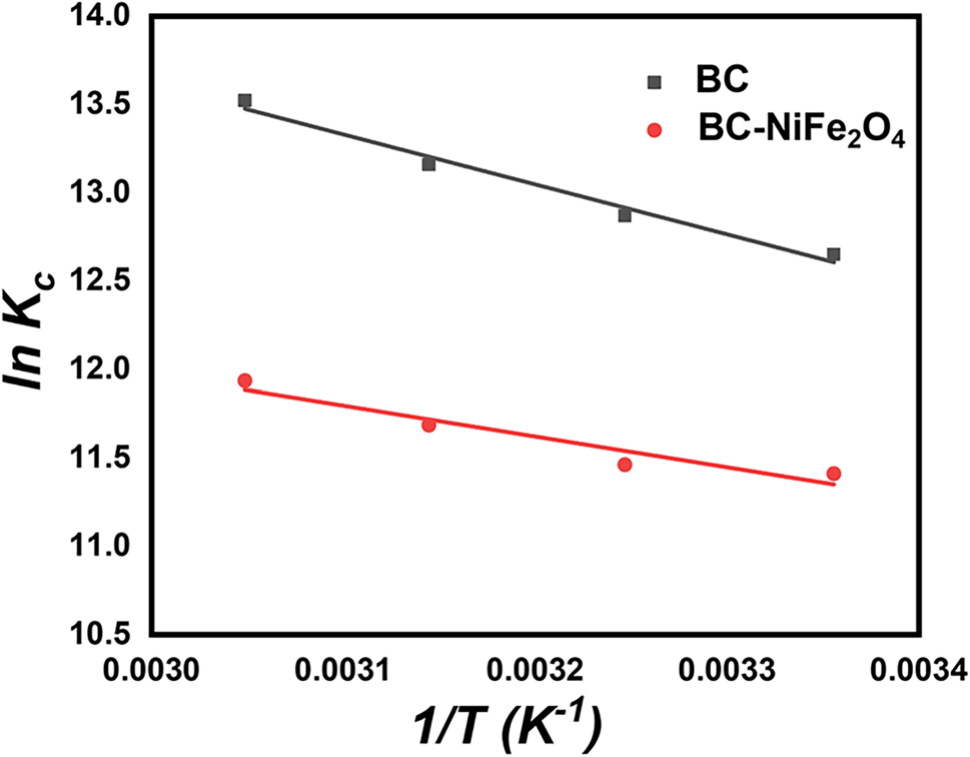
Thermodynamic behaviors of CIP adsorption onto BC, BC-NiFe_2_O_4_ adsorbents

Understanding the adsorption mechanism is greatly facilitated by studying adsorption kinetics. Four different kinetic models based on the results of experiments were conducted to investigate the impact of contact time on the adsorption process. These models are Pseudo-first-order, pseudo-second-order (Li et al. 2020), Elovich (Ngakou et al. 2019), and the intraparticle diffusion (Genç and Dogan 2015), Eqs. (3–6), respectively. In addition, the RMSE (Root Mean Square Error) and χ*2* (mathematically error) are used to measure the correlation between experimental data and theoretical models as shown in Eqs. (7 and 8).

3 $${q}_{t }={q}_{e}(1+{e}^{-{k}_{1}t})$$

4 $${q}_{t }=\frac{{{q}_{e}}^{2}\times t\times {k}_{2}}{1+{k}_{2}\times {q}_{e}\times t}$$

5 $${q}_{\mathrm{t}}=\frac{1}{\beta } \mathrm{ln}(\alpha \beta t+1)$$

6 $${q}_{\mathrm{t}}={ K}_{diff} {\mathrm{t}}^{0.5}+L$$

7 $$RMSE=\sqrt{\frac{1}{(n-1)}\sum_{n-1}^{n} ({q}_{e,exp}-{q}_{e, Cal})2}$$

8 $$\upchi 2=\sum_{n=1}^{n}\frac{({q}_{e, exp}-{q}_{e,Cal})2}{{q}_{e,Cal}}$$

where *q*_e_ (mg g^−1^) represents the quantity of CIP removed per adsorbent weight at equilibrium and *q*_t_ (mg/g) represents the quantity of CIP adsorbed per adsorbent weight at time *t*; *k*_1_ (min^−1^) and *k*_2_ (g mg^−1^ min) represent the rate constant of pseudo-first-order and pseudo-second-order rate constant (Eqs. 3, 4), repectively; *α* is initial adsorption rate (mg/g min), *β* is desorption constant (g mg^−1^); *K*_*diff*_ (mg g^−1^ min ^−0.5^) is the intraparticle diffusion constant, and *L* (g mg^−1^) represents the layer thickness. Figure 5 and Table 2 display the plots of kinetic models and their nonlinear parameters. As shown in Table 2, the coefficient of determination *R*^2^ values estimated from the pseudo-first-order model (≤ 0.439) was considerably lower than those calculated from the pseudo-second-order model (0.916), respectively. Moreover, the χ*2* and RMSE values estimated using the pseudo-second-order model are found to be the smallest. As a result, the pseudo-second-order model (Fig. 5a) was a better fit to predict the adsorption of CIP onto the two adsorbents, which has been suggested to be chemisorption due to the excellent goodness between actual and predicted values. The Elovich model (Fig. 6S) describes the kinetics of chemisorption on a heterogeneous surface adsorbent, and Eq. 5 is used to interpret the experimental result (Ngakou et al. 2019). In addition, Table 2 shows that the Elovich model fits the kinetic data well, indicating that the adsorption of CIP antibiotic onto BC and BC-NiFe_2_O_4_ is a heterogeneous diffusion process rather than a first-order reaction. In addition, the intra diffusion model (Fig. 5b) can provide detailed information regarding the mass transfer process of CIP during adsorption. Obviously, the *L* value calculated from the intra diffusion model is not equal to zero. As a result, one might deduce that CIP removal occurred through a different mechanism. As a result, intra-particle diffusion also fit to further elucidate the kinetics and rate-limiting step in the adsorption of CIP. The entire adsorption phenomenon comprises two major steps: (i) external diffusion, where the CIP transfers from the solution to the surface of adsorbent; (ii) intra-particle or pore diffusion, where CIP diffusion transfer from the external surface of the adsorbents to the inner pores. It seems that the straight line from the origin diverges, which indicates that there are additional rate-controlling steps besides intraparticle diffusion including metal-antibiotic complex.

**Table 2:** Kinetic parameters of CIP adsorption onto BC, BC-NiFe_2_O_4_ adsorbents

Kinetic model		Adsorbents	
		BC	BC-NiFe_2_O_4_
Pseudo-first order	*R*^2^	0.349	0.456
	*K* (min ^−1^)	0.343	0.439
	*χ2*	1.65	0.505
	*RMSE*	1.28	0.710
Pseudo-second order	*R*^*2*^	0.999	0.916
	k (g mg^−1^ min ^−1^)	0.033	0.058
	*q*_*e*_* exp* (mg g^−1^)	20.97	21.45
	*q*_*e*_* cal* (mg g^−1^)	22.22	21.73
	*χ2*	0.646	0.130
	*RMSE*	0.804	0.361
Elovich	*R*^*2*^	0.950	0.906
	*A*_*E*_ (g mg^−1^)	19785.7	2.69 × 108
	*B*_*E*_ (mg (g min^−1^)	0.659	1.11
	*χ2*	0.125	0.086
	*RMSE*	0.353	0.294
Intraparticle diffusion	*R*^*2*^	0.915	0.763
	*K*_*diff*_ (mg g^−1^ min^−1/2^)	0.539	0.301
	*χ2*	0.215	0.220
	*RMSE*	0.463	0.469

### Isotherm studies

**Fig. 7:**
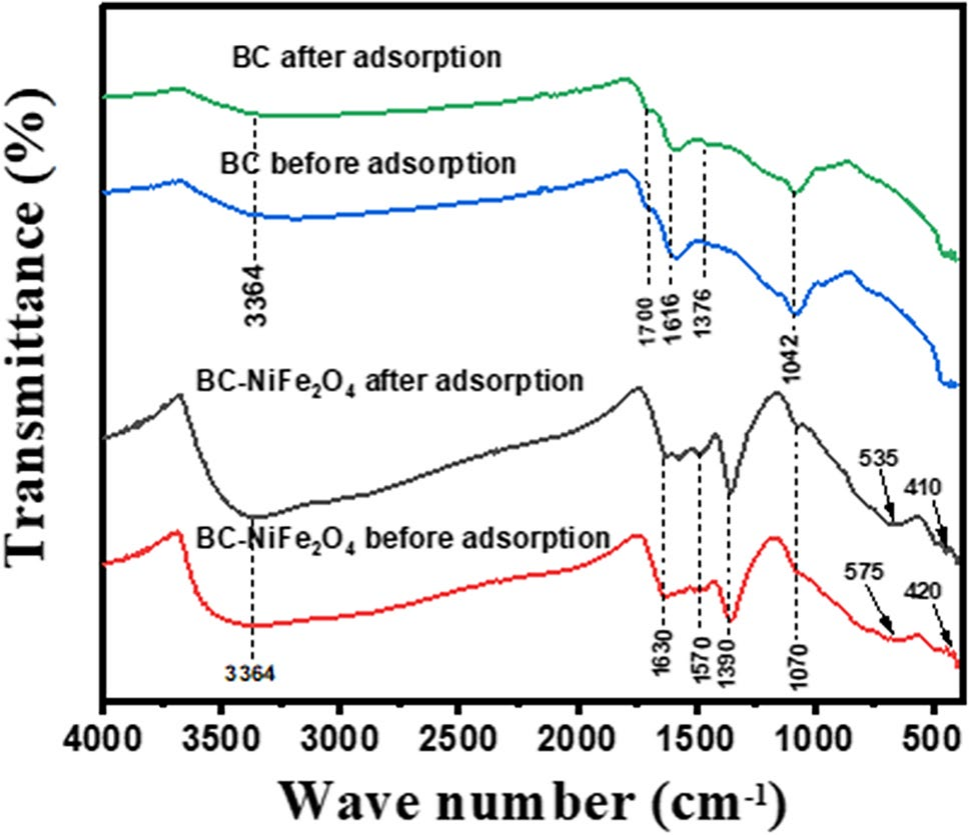
FTIR spectra of BC and BC-NiFe_2_O_4_ before and after adsorption of CIP

The adsorption isotherm relates the equilibrium concentration of CIP molecules in the solution to the amount of CIP antibiotic that is adsorbed per unit weight. The Langmuir model (Fig. 5c) is well-known for assuming monolayer adsorption on adsorbents with a homogeneous dispersion of adsorption sites, whereas the Freundlich model (Fig. 7S) is better suited for characterizing heterogeneous surface adsorption (Heo et al. 2019; Chakhtouna et al. 2021; Hu et al. 2021)**.** The Temkin isotherm (Fig. 7S) suggests that the heat of adsorption of all molecules decreases with the molecular coverage of the adsorbent surface and the adsorption process is homogeneously distributed. Dubinin-Radushkevich (D-R) isotherm (Fig. 7S) explains the free energies of the adsorption (Foo and Hameed 2012; Ashiq et al. 2019; Rahdar et al. 2019; Mahmoud et al. 2021).

Equations (9–12) represent the Langmuir, Freundlich, Temkin, and D-R isotherms, respectively (Bonilla-Petriciolet et al. 2017; Li et al. 2020; Rodrigues et al. 2020).

9 $${q}_{e}=\frac{{q}_{m}\times {K}_{L}\times {C}_{e}}{{1+\mathrm{K}}_{\mathrm{L}}{\mathrm{C}}_{\mathrm{e}}}$$

10 $${q}_{e}={K}_{f}\times {C}_{e}^\frac{1}{n}$$

11 $${q}_{e}=\mathrm{Bln}({\mathrm{K}}_{\mathrm{T}}{\times C}_{e})$$

12 $${q}_{e}={q}_{D-R}\times {e}^{-\upbeta {\upvarepsilon }^{2}}$$

12.1 $$\varepsilon =\mathrm{RTln }(1+\frac{1}{{C}_{\mathrm{e}}})$$

12.2 $${E}_{\mathrm{S}}=\frac{1}{\sqrt{2\upbeta }}$$

where *q*_*e*_ (mg g^−1^) is the amount of adsorbed CIP molecules per adsorbent materials weight; *C*_*e*_ (mg L^−1^) represents the residual of CIP. The maximum quantity of removal is represented by q_*m*_ (mg g^−1^); the Langmuir and Freundlich constants are denoted by *K*_*L*_ (L/mg) and *K*_*f*_ (mg g^−1^), respectively; (1/*n*) represents the adsorption intensity factor. *K*_T_ (L g^−1^) is the binding constant of Temkin; *B* (RT/*b*_T_) is the heat generated from adsorption (J mol^−1^); *b*_T_ is Temkin constant; *q*_*D-R*_ represents an isotherm saturation capacity; *β* is D-R isotherm constant; ε is the Polanyi potentials (J/mol); and *E*_s_ mean free energy (KJ mol^−1^); real gas constant (8.314 J mol^−1^ K^−1^) and temperature of solutions are denoted by* R* and *T*(K), respectively. The *R*^2^, χ*2*, and RMSE values for the adsorption of CIP on the BC and BC-NiFe_2_O_4_ show that the adsorption process effectively matches the Langmuir model (Fig. 5c, Table 3). The adsorption capacity of the BC-NiFe_2_O_4_ heterostructure was 68.79 mg g^−1^ compared to the BC sample (35.71 mg g^−1^) and NiFe_2_O_4_ nanoparticles (26.67 mg g^−1^). Moreover, BC-NiFe_2_O_4_ exhibited an efficient adsorption capacity compared to our previous work (Azzam et al. 2022). The *b*_*T*_ values of BC and BC-NiFe_2_O_4_ were 42.97 and 6.82 kJ mol^−1^, respectively, demonstrating the endothermic nature of the adsorption process (El-Shafey et al. 2012). In addition, the mean free energy of the D-R model for BC and BC-NiFe_2_O_4_ was 2.29 and 0.707 kJ mol^−1^, respectively, which is smaller than 8 kJ mol^−1^. This results suggests that a physical process has a critical role in the adsorption of CIP onto BC and BC-NiFe_2_O_4_.

**Table 3:** Non-linear isotherm of the Langmuir, Freundlich, Temkin, and D-R models for the adsorption of CIP onto BC, BC-NiFe_2_O_4_ adsorbents

Isotherm parameters		Adsorbents	
		BC	BC-NiFe_2_O_4_
Langmuir model	*q*_*max*_ (mg g^−1^)	35.71	68.89
	*K*_*L*_ (L mg^−1^)	1.39	0.273
	*R*^*2*^	0.982	0.982
	*χ2*	2.54	8.16
	*RMSE*	1.59	2.85
Freundlich model	*K*_*f*_ (mg g^−1^)	16.77	20.26
	1*/n*	0.181	0.344
	*R*^*2*^	0.809	0.910
	*χ2*	27.94	42.20
	*RMSE*	5.28	6.49
Temkin model	*b*_*T*_ (KJ mol^−1^)	42.97	6.82
	*K*_T_ (L g^−1^)	4.50	11.22
	*R*^*2*^	0.893	0.926
	*χ2*	15.64	34.66
	*RMSE*	3.95	5.88
Dubinin–Radushkevich model	*q*_*D-R*_	21.71	27.16
	*Β*)mol^2^.(kj^2^)^−1^)	8.41 × 10 ^−5^	0.001
	*E*_*s*_ (KJ mol^−1^)	2.29	0.792
	*R*^*2*^	0.984	0.935
	*χ2*	13.24	31.81
	*RMSE*	11.50	17.8

### Thermodynamic study

Thermodynamic studies are useful for predicting the adsorption mechanisms of contaminants onto adsorbent materials. Several distinct types of thermodynamic metrics, such as enthalpy change (△*H*°), Gibbs free energy change (△*G*°), and entropy change (△*S*°), were evaluated to investigate the adsorption nature and spontaneity of CIP. As seen in Fig. 6, thermodynamic data were evaluated to depict how temperature affected the adsorption process. The impact of temperature was investigated in the range of 298 to 328 K. Herein, CIP antibiotic (40 mg L^−1^) was dispersed into 2 g L^−1^ of the BC or BC-NiFe_2_O_4_ samples at different temperatures (298, 308, 318, and 328 K) to study the thermodynamics of CIP removal using Van’t Hoff equations (Eqs. (13–16)).

13 $$\Delta G^\circ =-RT \mathrm{ln}{K}_{c}$$

where *K*_c_ (corrected Langmuir constant) can be determined from the Langmuir constant *K*_L_ as follows:

14 $${K}_{c}={M\times K}_{L} \times 1000$$

15 $$\Delta G^\circ =\Delta H^\circ -T\Delta S^\circ$$

16 $$\mathrm{ln} {K}_{\mathrm{c}}=\frac{\Delta S^\circ }{R}-\frac{\Delta H^\circ }{RT}$$

△*H°* and △*S°* values were derived using the slope and intercept of ln*K*_*c*_ against *1/T* (Fig. 6), and △*G°* was calculated by using Eq. (13). M (g/mol) is the molecular weight of the CIP, *R* (kJ mol^−1^ K^−1^) is the real gas constant (8.314 × 10^−3^), and *T* is the solution’s temperature (K). Table 4 displays the thermodynamic parametars of CIP removal onto the adsorbent samples. The standard Gibbs free energies “△*G°*” are negative values, suggesting that CIP adsorption was spontaneous and feasible (Emily Chelangat Ngeno et al. 2016). The adsorption process was more effective at higher temperatures due to the Δ*G*° value for CIP adsorption onto the surfaces of the two adsorbents decreased as temperature increased. Furthermore, the positive values of △*H°* indicate the endothermic character of the adsorption process. On the other hand, the positive △*S°* values suggested the increase in randomness at the solid–liquid interface.

**Table 4:** Thermodynamic parameters for CIP adsorption onto BC, BC-NiFe_2_O_4_ adsorbents using Langmuir isotherm data

Adsorbent	△*H*º (kJ mol^−1^)	△*S*º (J mol^−1^ K^−1^)	△*G*º (kJ mol^−1^)			
			298 K	308 K	318 K	328 K
BC	23.52	183.79	− 31.34	− 32.95	− 34.79	− 36.87
BC-NiFe_2_O_4_	14.47	142.96	− 28.27	− 29.35	− 30.89	− 32.55

### Proposal interpretation of the CIP adsorption mechanism

#### FTIR analysis

FTIR analysis (Fig. 7) was utilized to elucidate the adsorption mechanism because it is efficient at examining the functional groups of the adsorbents. The changes in the functional groups before and after the CIP removal is believed to be a strong indicator of the adsorption of adsorbate on the surface of BC and BC-NiFe_2_O_4_. The peak at 3364 cm^−1^ was attributed to the O–H stretching vibrations and responsible for water sensitivity or hydrophilicity. The C = C stretching and the carbonyl group (C = O) in the benzene rings in the BC sample were attributed to the vibrational peaks at 1616 and 1700 cm^−1^, respectively. These results showed relatively stable aromatic or graphitic structures that are favorable for contaminants’ adsorption through π-π interactions (Nogueira et al. 2018; Hu et al. 2021). Moreover, the peaks at 1376 cm^−1^ show the existence of C–O stretching vibration in the carboxylate groups. Furthermore, the existence of C-O stretching in the alcohol functional groups is demonstrated by peaks at 1042 cm^−1^ that shift to 1070 cm^−1^ in BC-NiFe_2_O_4_ because of the formation of H-bonding. The FTIR spectra of BC-NiFe_2_O_4_ showed two distinct peaks at 575 and 420 cm^−1^ (Ni–O-Fe and Fe–O) (Livani and Ghorbani 2018; Livani et al. 2018; Taj et al. 2021), indicating that the NiFe_2_O_4_ was constructed on the surface of biochar, which shifted to 535 and 410 cm^−1^ after CIP adsorption because of the metal coordination reactions. These results, which are consistent with the XRD analysis, confirm the successful preparation of BC-NiFe_2_O_4_ adsorbent (Aliahmad et al. 2013; Gor and Dave 2020). The peak located at 1390 cm^−1^ is related to the stretching C = O of ketones and quinones groups (Chakhtouna et al. 2021). Moreover, the stretching vibration of the C = C band of the aromatic ring in the BC-NiFe_2_O_4_ sample was assigned at 1570 cm^−1^ to 1630 cm^−1^ (Patel et al. 2021a). Therefore, these results confirm that the coordination reaction and π-π stacking further supported the adsorption mechanism between CIP and BC-NiFe_2_O_4_.

#### XPS analysis

**Fig. 8:**
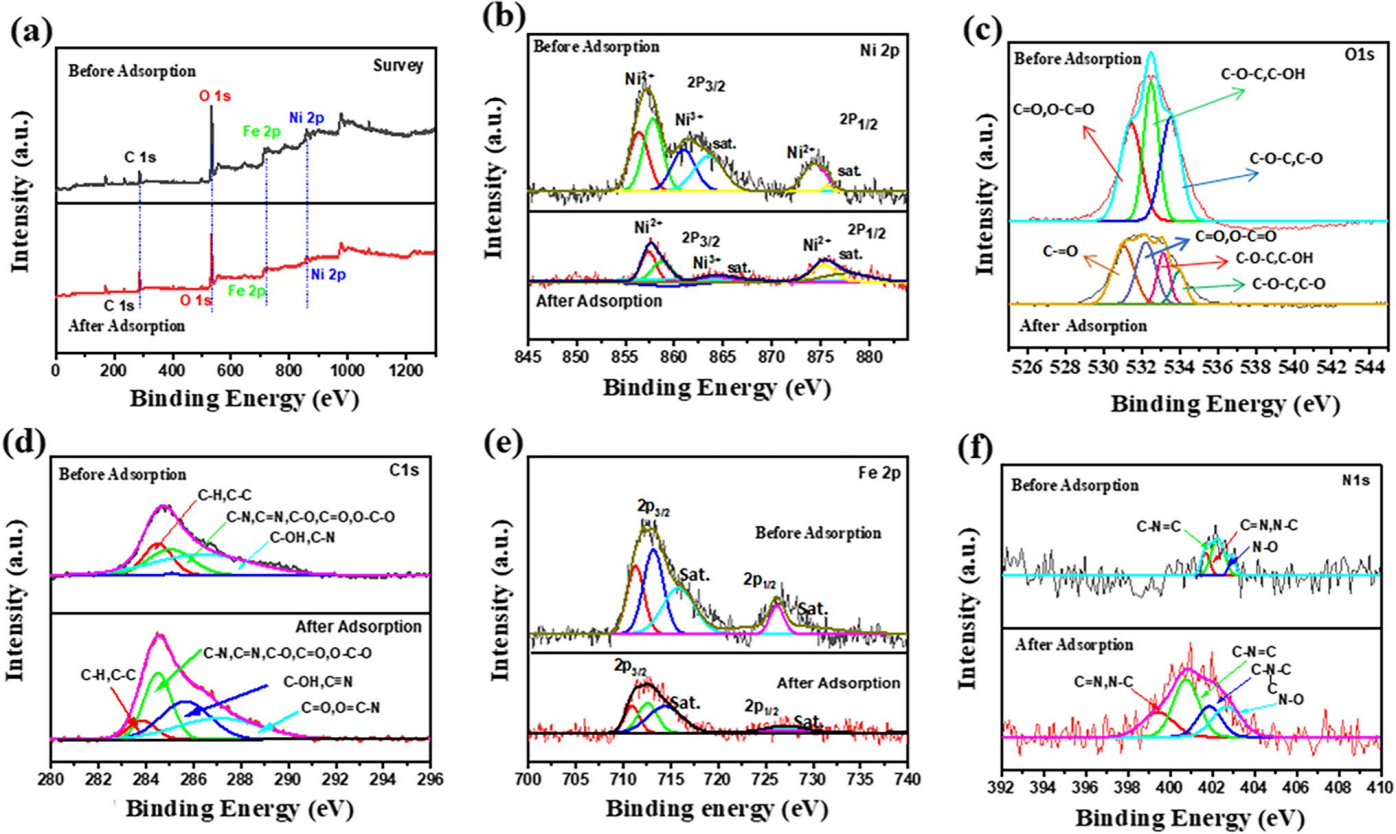
XPS spectra of BC- NiFe_2_O_4_ hybrid structure before and after adsorption of CIP: **a** full survey, **b** Ni 2p, **c** O 1 s, **d** C 1 s, **e** Fe 2p, and **f** N 1 s

**Fig. 9:**
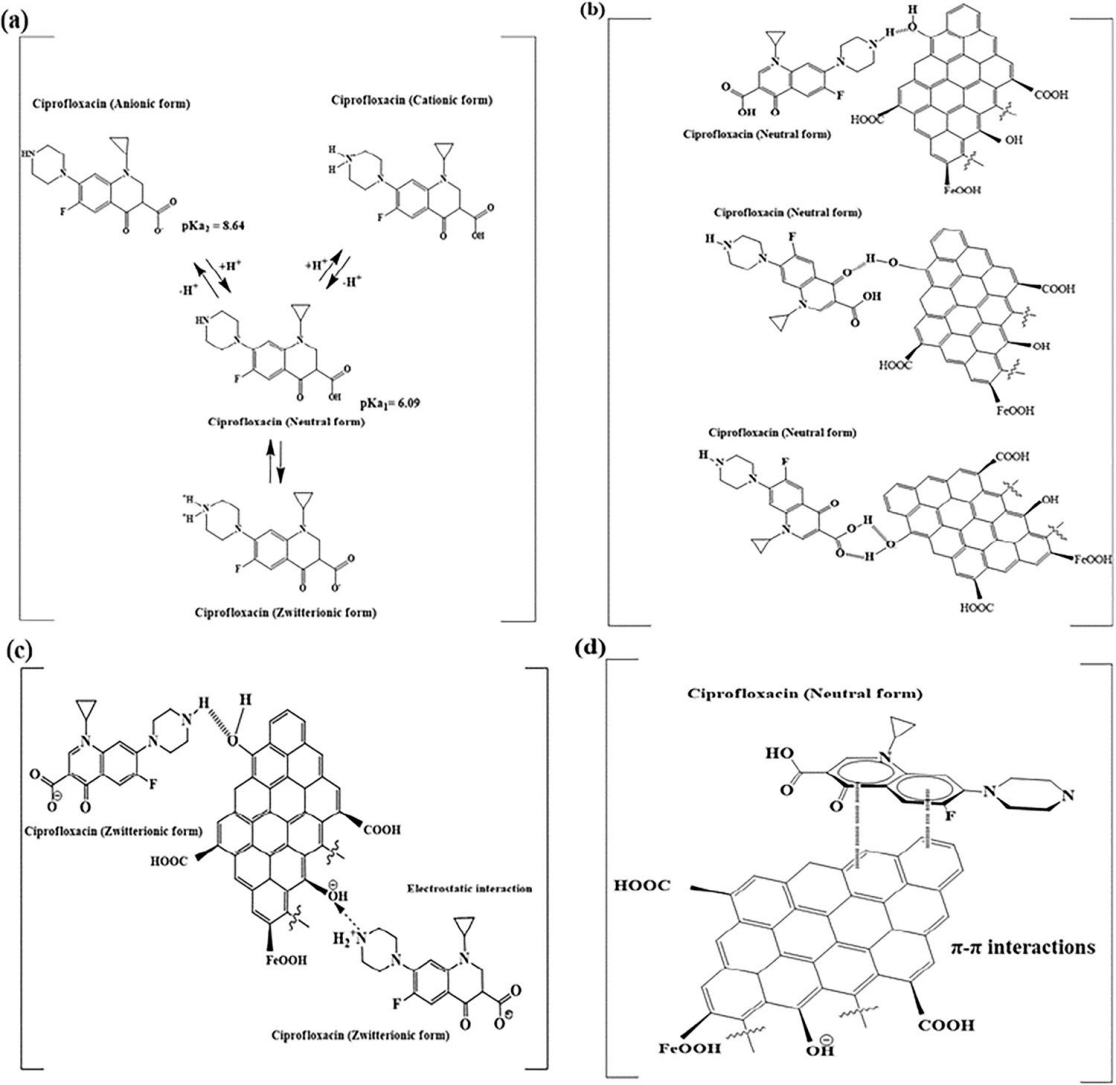

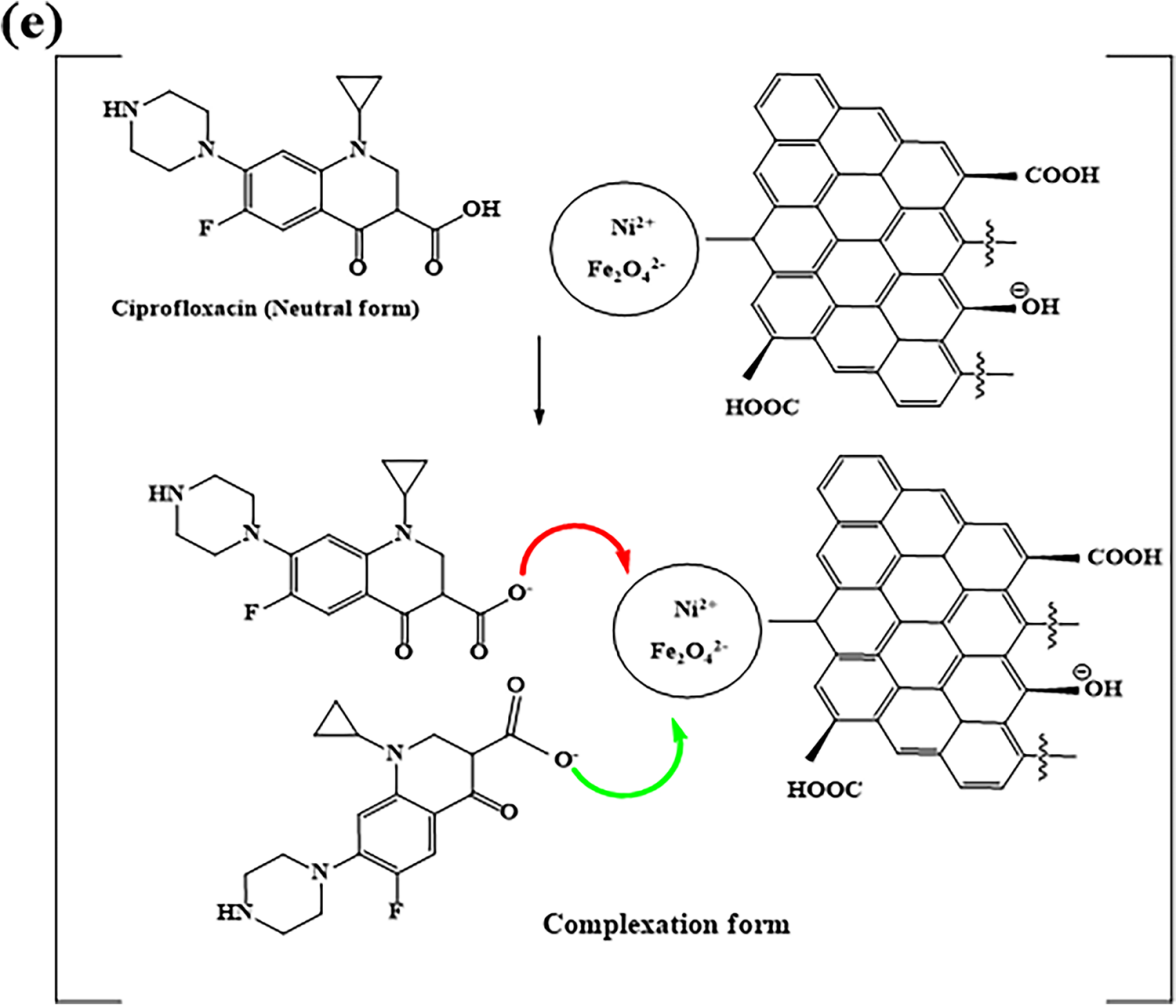
Possible CIP adsorption mechanisms. **a** Speciation of CIP, (**b**) H-bonding between CIP zwitterion and biochar’s functional groups, **c** H-bonding between CIP zwitterion and biochar’s carbonyl groups, **d** possible π-π interactions between CIP and biochar, and **e** complexation reaction between metal-biochar and CIP

Figure 8a shows the BC-NiFe_2_O_4_ XPS spectrum before and after the CIP adsorption. The XPS spectrum (Fig. 8a) shows clearly the characteristic peaks of Ni, O, C, Fe, and N elements, no impurity peaks were detected. The XPS spectrum of Ni 2p showed peaks for Ni 2p_3/2_ and Ni 2p_1/2_ (Fig. 8b). The peaks in the spin-orbital Ni 2p_3/2_ that are assigned to Ni^2+^ and Ni^3+^ have binding energies of 856.2, 861.6, 865.1, and 874.9 eV. The binding energies of the satellite peaks, denoted by the prefix “sat,” are 867.7 eV and 878.1 eV, respectively (Hua et al. 2018; Iraqui et al. 2020). Furthermore, the Ni 2p_3/2_ peaks were shifted to lower binding energies of 856.0, 861.7, 864.9, and 874.7 eV, and the intensity of all peaks were reduced, demonstrating that metallic coordination may be formed with CIP (Fig. 9e). Figure 8c shows the O 1 s core level spectrum, which had peaks at 531.3, 533.1, and 534.2 eV (Li et al. 2018a; Wu et al. 2021). The peak at 531.3 eV is related to the -C = O and O-C = O groups, whereas the peak at 533.1 eV is attributed to C–O–C and C–OH groups. Finally, the peak at 534.2 eV is assigned to the C-O group (Hua et al. 2018; Li et al. 2018a; Feng et al. 2021). The peak found at 530.8 eV after CIP adsorption was assigned to the carbonyl group. The observed substantial decrease in peak intensities 531.3 and 534.2 eV can also be attributed to the hydrogen bonding interactions between the O-C = O, C–OH groups and CIP molecules (shown in Fig. 9a). The XPS spectrum of C1s at a binding energy of 284.8 eV (Fig. 8d) is related to the C–C = C, C–C, and C-H groups of organic compounds. These organic compounds are appropriate for binding the CIP through π-π reaction (Fig. 9d). Moreover, the peak at 285.1 eV is ascribed to the C-O groups (Chowdhury et al. 2021). The binding energy for C–OH or C≡N groups is 286.72 eV (Yan et al. 2021). After CIP adsorption on biochar/nickel ferrite nanocomposite, a new peak appeared at 287.91 eV ascribed to the carbonyl groups. The Fe 2p core-level XPS spectrum (Fig. 8e) exhibits two broad peaks at 711.7 eV and 724.8 eV, which are assigned to Fe 2p_3/2_ and Fe 2p_1/2_, respectively, with a satellite peak of Fe 2p_3/2_ at 718.1 and 730.3 eV. These peaks confirm the existence of Fe (III) in the prepared adsorbent (BC-NiFe_2_O_4_) (Singh Yadav et al. 2015; Hua et al. 2018). Whereas, CIP loaded BC-NiFe_2_O_4_ shifted iron to lower binding energies of 710.3 eV and 723.6 eV, indicating that a metal-antibiotics complex may be formed (Fig. 9e). The N 1 s core level spectra is shown in Fig. 8f, with three peaks at 398.35, 400.41, and 401.98 eV. These peaks are related to N≡C group, C-N–C group, and the N–O group, respectively. After CIP adsorption, a new peak at 402.2 eV appeared, which is assigned to the quaternary N atom. The intensity of all peaks was decreased due to either hydrogen bonding or electrostatic interactions between the C-O on the adsorbent surface and the NH_2_^+^ in the ionic species of the CIP (Fig. 9b–d). Therfore, biochar contained a number of additional functional groups, such as C–O–C, C = O, C-N, C = N, and C–OH which could form a hydrogen bond with the CIP structure (-NH-, -COOH, and -F) and increase the CIP removal. In addition, the removal of CIP by BC and BC-NiFe_2_O_4_ based adsorbents is not only dependent on the pore volume, and pore structure, but is also influenced by other parameters such as coordination affinities, π-π stacking, and H-bonding interactions that confirmed by FTIR and XPS analysis. Although, the total pore volume (*V*_*T*_*)* of BC and BC-NiFe_2_O_4_ reduced from 0.553 to 0.201 cm^3^ g^−1^, respectively, but the removal % was enhanced, confirming other key factors affecting the adsorption process. This finding indicated that coordination affinities, π-π stacking, and H-bonding interactions demonstrated the dominant adsorption mechanisms.

### *Environmental application of BC-NiFe*_*2*_*O*_*4*_* for CIP removal*

The efficiency of BC-NiFe_2_O_4_ heterostructure as a CIP adsorbent was evaluated using three water samples collected from Helwan City, Egypt, including Nile water, groundwater, and pharmaceutical wastewater. A 0.1 g of BC-NiFe_2_O_4_ was mixed and spiked with 50 mL of each water sample with the initial concentration of 10 mg L^−1^ CIP. The initial pH values of solutions were adjusted to pH 6.0. The removal percentages are shown in Table S2. The CIP removal efficiency for Nile, ground, and pharmaceutical wastewater were 62.12%, 60.98%, and 93.81%, respectively. However, when an aqueous solution sample was used, it was 99.33%. The decrease in removal percentage can be explained by the presence of interfering ions.

### *Evaluation of reusability and comparison of BC-NiFe*_*2*_*O*_*4*_* removal efficacy with different adsorbents*

**Fig. 10:**
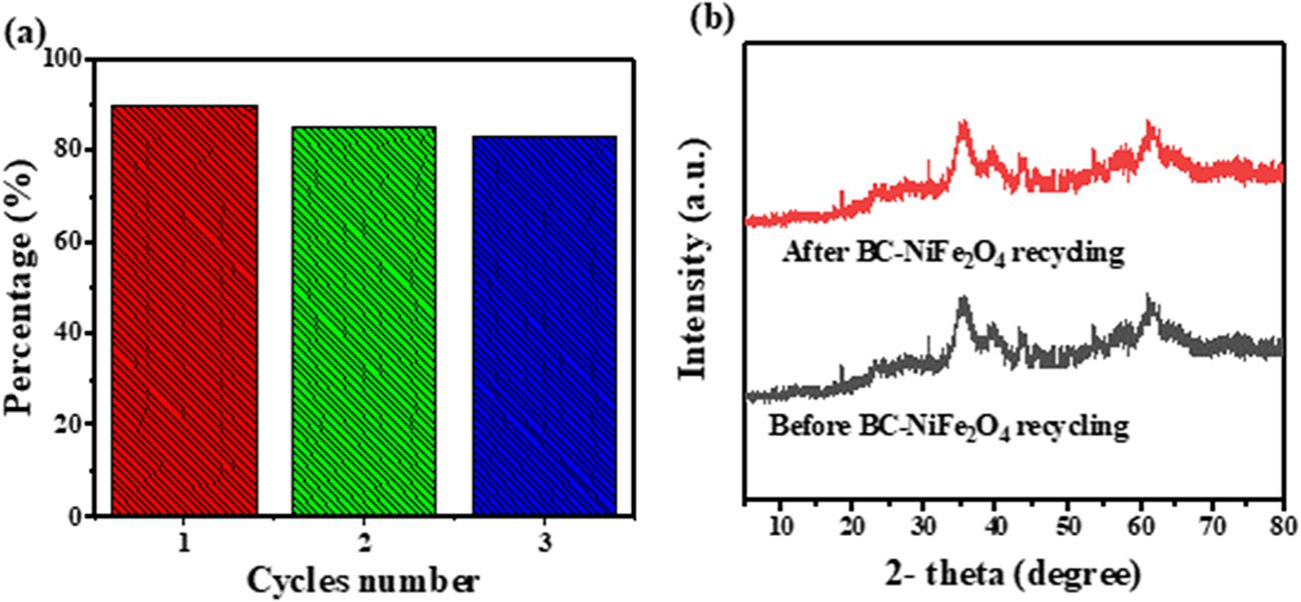
**a** Cycling times on the BC-NiFe_2_O_4_ hybrid structure for CIP removal and **b** XRD patterns for BC- NiFe_2_O_4_ hybrid structure before and after the third recycle

The reusability of BC-NiFe_2_O_4_ composites was evaluated using 0.1 M sodium hydroxide (NaOH) as a stripping agent. In a batch experiment, BC-NiFe_2_O_4_-loaded CIP molecules were agitated in 50 mL of 0.1 M sodium hydroxide (NaOH) for 30 min. After regeneration, the adsorbent was washed several times with distilled water to remove the excess NaOH and dried at 80 °C to use in the next cycle. The removal efficiency of CIP after three cycles remained at 83.79%, as shown in Fig. 10a. Furthermore, as illustrated in Fig. 10b, the XRD analysis before and after regeneration has the same pattern, confirming the stability of BC-NiFe_2_O_4_ nanocomposites. Furthermore, the maximal adsorption capacity for CIP onto BC-NiFe_2_O_4_ via the Langmuir adsorption model was found to be 68.79 mg g^−1^, which is higher than previously reported adsorbents in the literature (Table 5). Therefore, BC-NiFe_2_O_4_ has a potential candidate for commercial application from pharmaceutical wastewater.

**Table 5:** Comparison of the adsorption capacity of BC-NiFe_2_O_4_ for CIP removal with previously reported adsorbents

Adsorbent	*q*_max_ (mg g^−1^)	pH	Temp (°C)	Method of determination	Ref
Humic acid coated magnetic biochar	8.68	-	15	Langmuir	(Zhao et al. 2019)
Manganese oxide–loaded magnetic biochar (MMB)	8.37	3.0	***25***	Langmuir	(Li et al. 2018c)
ZnO nanoparticles	***8.30***	***6.8***	***25***	Langmuir	(Dhiman and Sharma 2019)
Modified coal fly ash	1.54	-	25	Langmuir	(Huang et al. 2014)
Nano graphene oxide-magnetite	2.2	6.5	21	Langmuir	(Alicanoglu and Sponza 2017)
Reduced graphene oxide/magnetic composite	18.98	6.2	25	Langmuir	(Tang et al. 2013)
Alkali activated potato stems and leaves biochar	6.62	-	15	Langmuir	(Li et al. 2018b)
Chitosan/biochar hydrogel beads (CBHB)	34.90	3.0	30	Langmuir	(Afzal et al. 2018)
Montmorillonite-cellulose acetate	13.8	6.5	25	Langmuir	(Das et al. 2020b)
MgO nanoparticles	3.47	6.0	-	Langmuir	(Khoshnamvand et al. 2017)
Biochar (herbal residue)	37.6	7	25	Langmuir	(Shang et al. 2016)
Pomegranate peel	8.05	6	25	Langmuir	(S.A Hassan and F.J.Ali 2014)
Black tea leaves	12.73	6	25	Langmuir	(S.A Hassan and F.J.Ali 2014)
Sawdust	11.6	5.8	25	Langmuir	(Bajpai et al. 2012)
Biochar from potato stem and leaves	23.36	-	35	Langmuir	(Li et al. 2018b)
Carbon nanofiber	10.36	5.0	25	Langmuir	(Li et al. 2016)
Biochar from water hyacinth	2.717	4.0	25	Langmuir	(Emily Chelangat Ngeno et al. 2016)
Water hyacinth biochar (WHC 350)	2.72	-	25	Langmuir	(Emily Chelangat Ngeno et al. 2016)
Potato stems and leaves biochar	10.13	-	15	Langmuir	(Li et al. 2018b)
CoFe_2_O_4_/Ag_2_O nano-composite	***40***	***-***	***25***	Langmuir	(Das et al. 2020a)
BC-NiS	41.66	6	25	Langmuir	(Azzam et al. 2022)
BC-NiFe_2_O_4_	68.7	6	25	Langmuir	This study

## Conclusions

A highly efficient and stable BC-NiFe_2_O_4_ nanocomposite having magnetic properties was successfully developed via a facile co-precipitation approach for the elimination of ciprofloxacin (CIP) from pharmaceutical wastewater. The loading of magnetically NiFe_2_O_4_ nanoparticles on the surface of porous biochar enhanced the stability and adsorption performance for CIP removal compared to our previous work on biochar decorated with NiS (BC-NiS). The characterization techniques proved that the desired adsorbents had been successfully synthesized. Significantly, BC-NiFe_2_O_4_ showed the greatest adsorption capacity (68.79 mg g^−1^) compared to BC (35.71 mg g^−1^). Moreover, the removal of CIP using BC-NiFe_2_O_4_ was improved up to 99.3% within 30 min without pH adjustment. The results indicated that the adsorption process fits the Langmuir isotherm model, pseudo-second-order kinetics, and its endothermic nature. The removal effecieny of BC-NiFe_2_O_4_ adsorbent decreased slightly after three consecutive cycles, confirming the chemical stability of heterogenous strurcure. The FTIR and XPS analysis proved that the π-π interaction, the hydrogen bond and coordination affinities were identified as the primary driving forces of the adsorption mechanism of BC-NiFe_2_O_4_. These results showed that loading NiFe_2_O_4_ nanoparticles onto porous biochar has the potential to be a low-cost adsorbent for water remediation due to its easy manufacturing process, high removal effectiveness, and recyclable nature.

### Supplementary Information

Below is the link to the electronic supplementary material.

Supplementary file1 (DOCX 1098 KB)

## Data Availability

All data generated or analyzed during this study are included in this published article.
