# Supplementary material for: Heterogeneous porous biochar-supported nano NiFe2O4 for efficient removal of hazardous antibiotic from pharmaceutical wastewater
Source: Environ Sci Pollut Res Int. 2023 Nov 6;30(56):119473–90. doi: 10.1007/s11356-023-30587-5 (PMC10698114; doi:10.1007/s11356-023-30587-5)
Supplement: Supplementary file 1 — Supplementary file1 (DOCX 1098 KB) [file 11356_2023_30587_MOESM1_ESM.docx]

**Supporting information**

**Heterogeneous porous biochar-supported nano NiFe_2_O_4_ for efficient removal of hazardous antibiotic from pharmaceutical wastewater**

Ahmed B. Azzam ^a^*, Yousif A. Tokhy ^a^, Farida M. El Dars ^a^, Ahmed A. Younes ^a^

^a*^ Chemistry Department, Faculty of Science, Helwan University, Ain Helwan, Cairo 11795, Egypt

***Corresponding authors:** Ahmed B. Azzam, [ahmed_azzam2000@hotmail.com](mailto:ahmed_azzam2000@hotmail.com), Tel.+201285259709

1. *Characterization methods*

X-ray diffraction (XRD; Bruker D8 Advance, X-ray diffractometer) was used to examine the crystalline structures of the three samples (BC, and BC- NiFe_2_O_4_). Field-emission scanning electron microscope at 20 kV (FSEM, Quanta 250 FEG) and a high-resolution transmission electron microscope (HRTEM, JEM-2100, JEOL, Japan) was used to examine the surface morphology of the materials. Brunauer–Emmett–Teller (BET, N2 @ 77K on silica (cyl. pore)(NLDFT Ads. model)) was utilize to determine the surface area of NiFe_2_O_4_. X-ray photon spectroscopy (XPS) was used to characterize the elemental composition and oxidation state, which was collected on the PHI Versa probe II with monochromatic Al Ka (15.0 kV with the kinetic energy of 1486.6 eV). FT-IR spectrometer Perkin Elmer (model spectrum one FT-IR spectrometer, USA) using standard KBr pellets between 4000 and 500 cm^-1^ was used to identify the functional groups on the adsorbents. Magnetic measurements were performed using (MPMS7XL) with a superconducting quantum interference device (SQUID) magnetometer (Quantum Design, USA).

1. *Materials*

In Cairo, Egypt, fresh bananas were purchased from a local supermarket. A local pharmaceutical manufacturer provided an analytical grade of CIP hydrochloride (Alex., Egypt). Stock solutions containing 1000 mg/L of CIP are prepared by dissolving CIP in double-distilled water. Nickel chloride (NiCl_2_) and ferric chloride (FeCl_3_) were provided from Sigma-Aldrich. The usage of all other reagents was unprocessed and they were all at least analytical grade when they were utilized


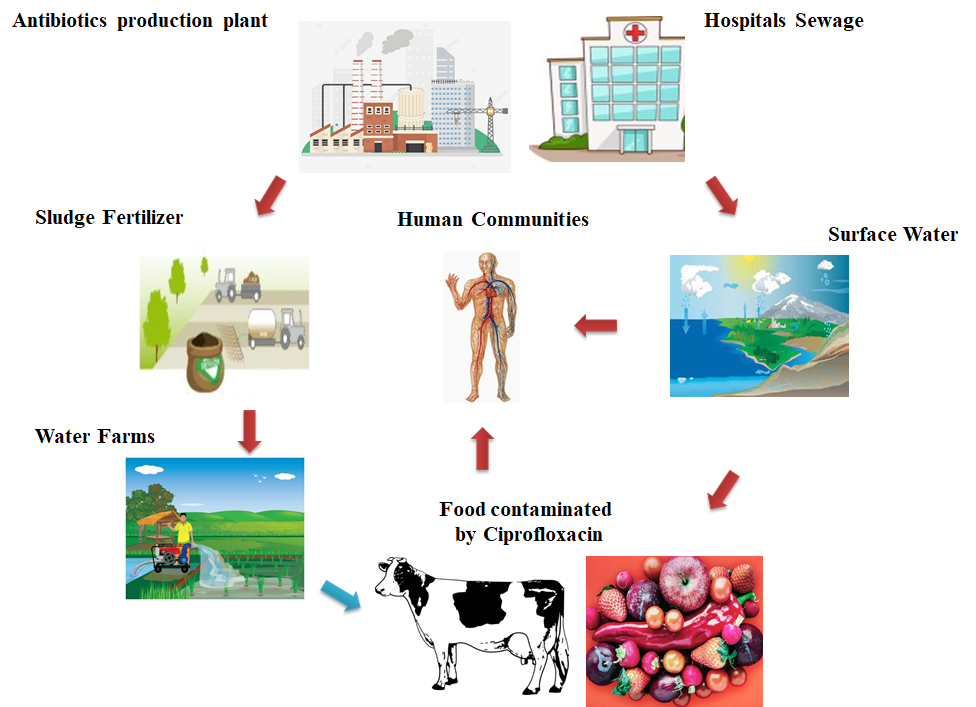


**Fig. S1.** Major pathways of antibiotics released into the environment


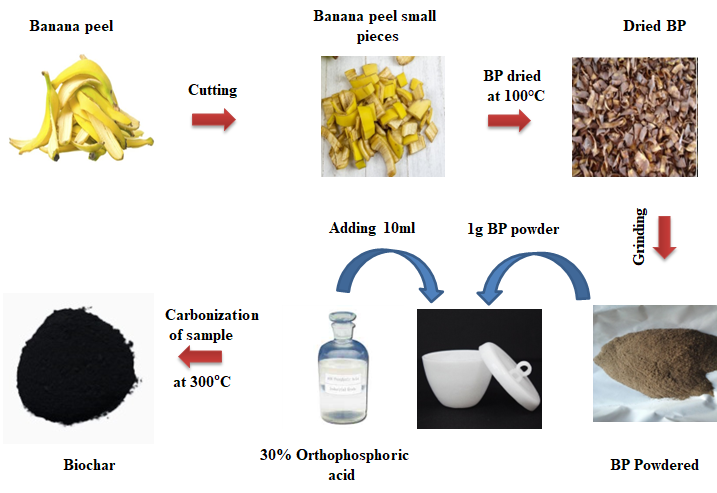


**Fig. S2.** Diagram representation of BC synthesis


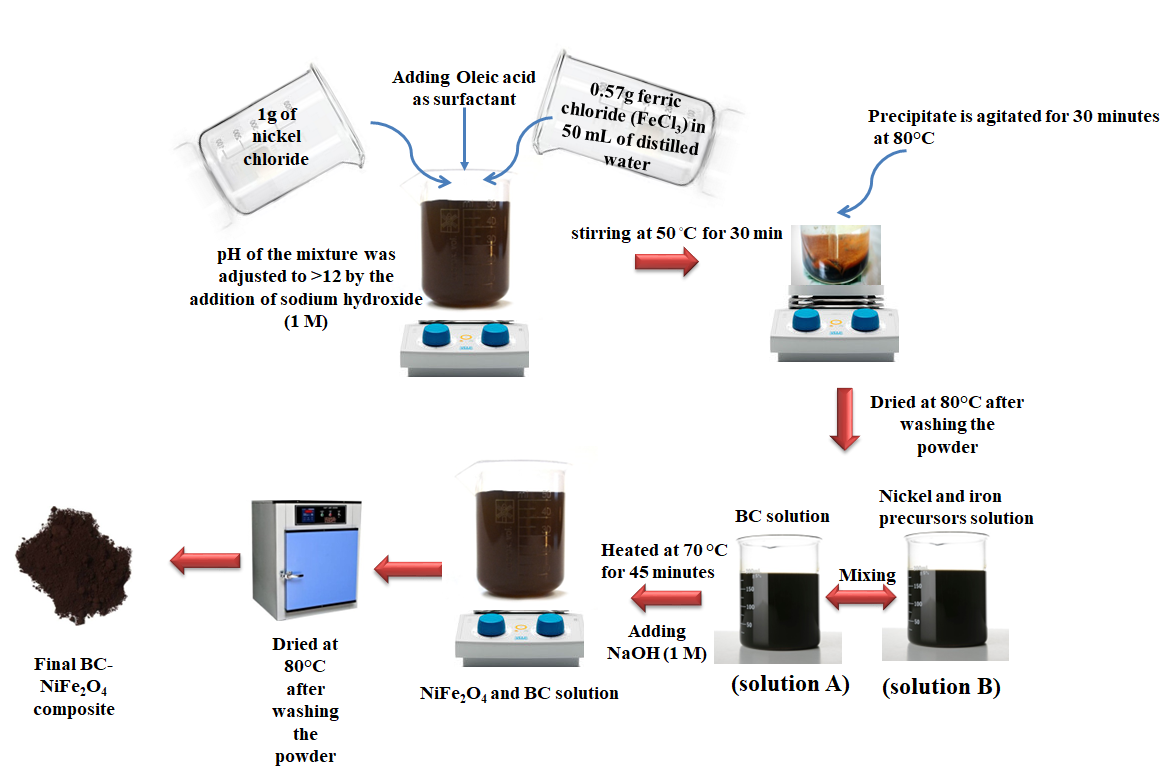


**Fig. S3.** Diagram representation of BC-NiFe_2_O_4_ synthesis


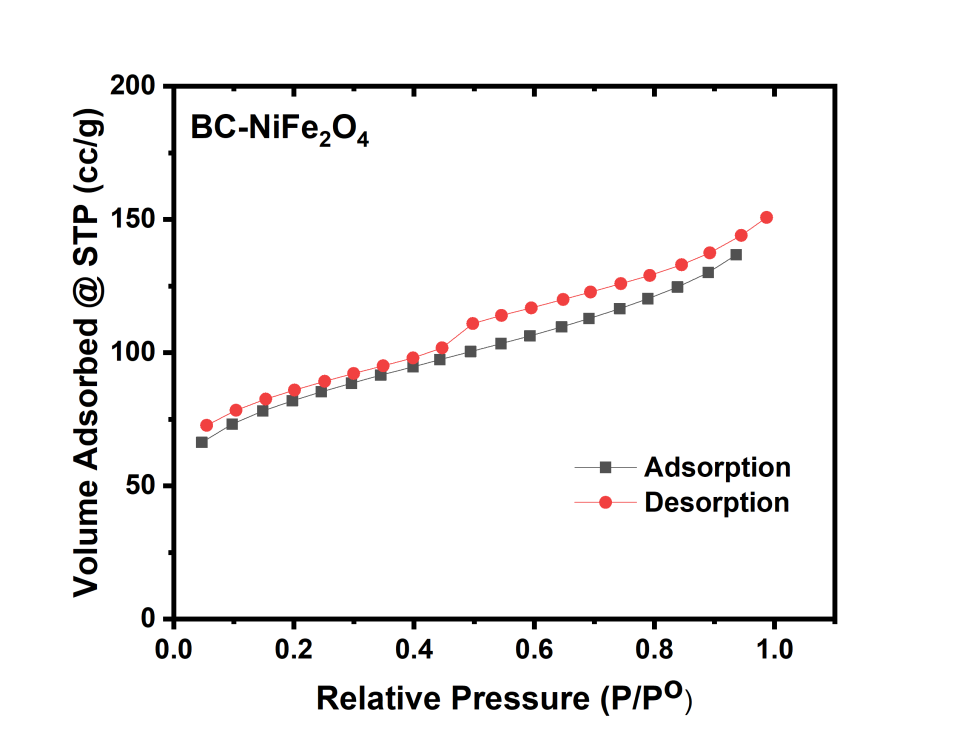

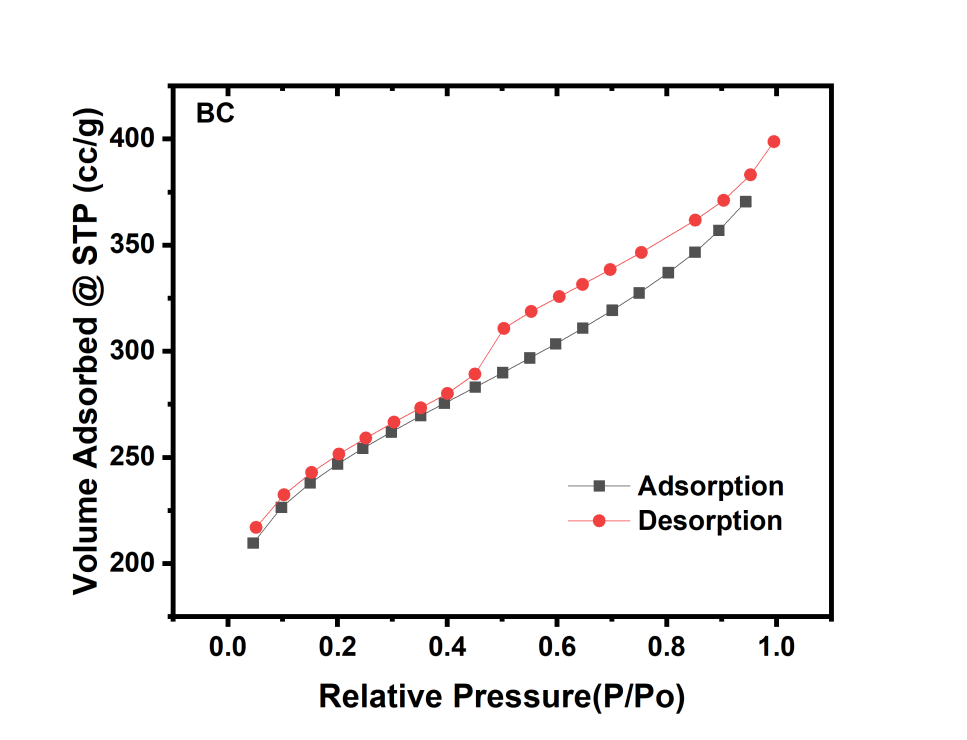


**Fig. S4. N2 adsorption–desorption isotherms of NiFe_2_O_4_**


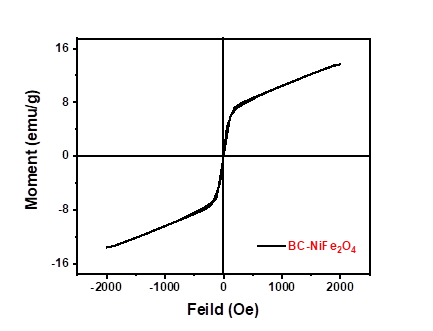


**Fig. S5.** Magnetization curve of BC-NiFe_2_O_4_

**Table S1** The magnetic properties of the prepared BC-NiFe_2_O_4_

| BC-NiFe_2_O_4_ | Ms (emu g^-1^) | Mr (emu/g) | Hs (Oe) | Hc (Oe) |
| --- | --- | --- | --- | --- |
|  | 10.02 | 5.26 | 52.03 | 8.21 |
| NiFe_2_O_4_ | 32.56 | 17.34 | 55.12 | 3.21 |

**Table S2** Analytical results for the removal of CIP from real water samples using BC-NiFe_2_O_4_

| Water samples | |  | Removal [%] |
| --- | --- | --- | --- |
| Nile water |  | | 62.12 |
| Groundwater |  | | 60.98 |
| Pharmaceutical wastewater |  | | 93.81 |


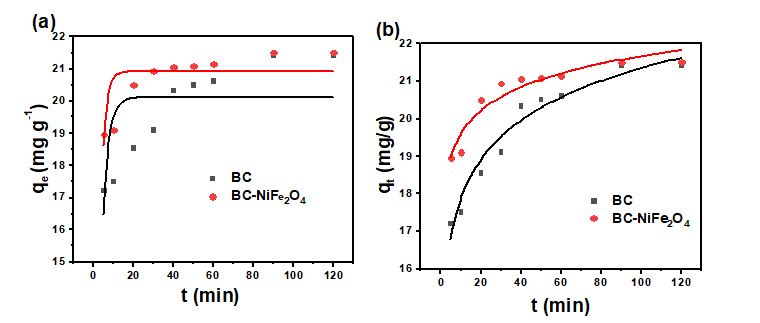


**Fig. S6** (a) pseudo-first-order plot; (b) Elovich; onto BC, and BC-NiFe_2_O_4_ adsorbents.


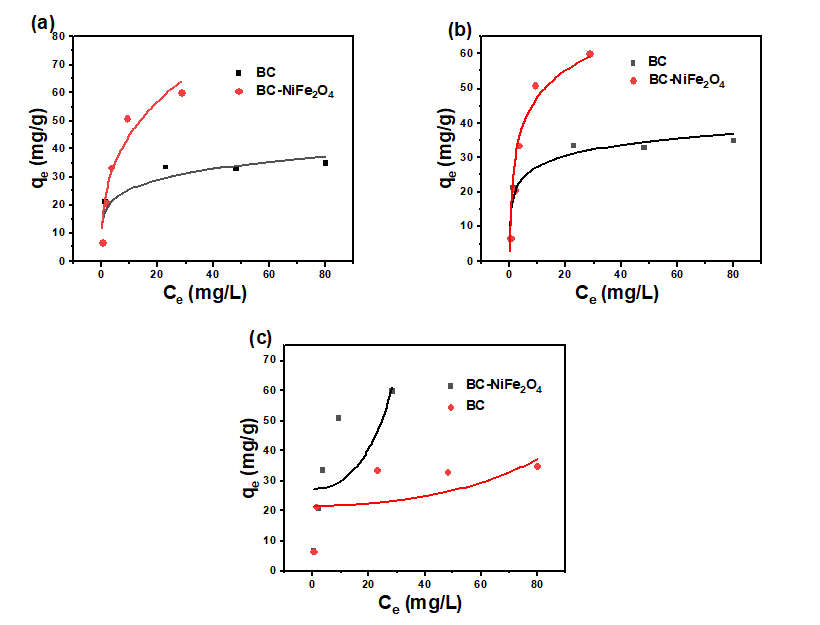


**Fig. S7** (a) Freundlich adsorption isotherms plot; (b) Temkin adsorption isotherms; (c) D-R adsorption isotherms plot onto BC, and BC-NiFe_2_O_4_ adsorbents
